# Targeting Neuroendocrine Prostate Cancer: Molecular and Clinical Perspectives

**DOI:** 10.3389/fonc.2015.00006

**Published:** 2015-02-02

**Authors:** Panagiotis J. Vlachostergios, Christos N. Papandreou

**Affiliations:** ^1^Department of Internal Medicine, Lutheran Medical Center, Brooklyn, NY, USA; ^2^Department of Medical Oncology, Faculty of Medicine, School of Health Sciences, University of Thessaly, Larissa, Greece

**Keywords:** neuroendocrine prostate cancer, small cell prostate carcinoma, targeted therapy, androgen-independent, castration-resistant

## Abstract

Neuroendocrine prostate carcinoma, either co-present with the local adenocarcinoma disease or as a result of transdifferentiation later in time, was described as one major process of emerging resistance to androgen deprivation therapies, and at the clinical level it is consistent with the development of rapidly progressive visceral disease, often in the absence of elevated serum prostate-specific antigen level. Until present, platinum-based chemotherapy has been the only treatment modality, able to produce a fair amount of responses but of short duration. Recently, several efforts for molecular characterization of this lethal phenotype have resulted in identification of novel signaling factors involved in microenvironment interactions, mitosis, and neural reprograming as potential therapeutic targets. Ongoing clinical testing of specific inhibitors of these targets, for example, Aurora kinase A inhibitors, in carefully selected patients and exploitation of expression changes of the target before and after manipulation is anticipated to increase the existing data and facilitate therapeutic decision making at this late stage of the disease when hormonal manipulations, even with the newest androgen-directed therapies are no longer feasible.

## Introduction

For over two decades, several efforts have been made to define and molecularly characterize the frame of neuroendocrine (NE) prostate cancer (NEPC) presenting with distinct clinical features, different from the classic prostatic adenocarcinoma, including frequent visceral metastases, lytic bone involvement, relative low serum prostate-specific antigen (PSA) concentration, resistance to androgen ablation, and high response rate to platinum-based chemotherapy (1). In the era of evolving androgen-directed therapies, better molecular characterization and targeting of the NE phenotype remains of central interest given the inherent or emerging resistance of NEPC cells to current therapies, abrogation of which is warranted in order to improve responses and mortality of prostate cancer (PC) patients.

## New Morphological Classification of NEPC

A new histological classification for NEPC has been recently proposed as a refinement of the current WHO morphologic criteria, to better reflect the aspects of NE differentiation in PC (2). According to the new classification, NEPC extends from usual NE differentiation found in usual prostate adenocarcinoma to mixed (small or large cell) NE carcinoma with acinar adenocarcinoma, adenocarcinoma with Paneth cell NE differentiation, carcinoid tumor, and small cell carcinoma. The new classification also satisfies the previously unmet need of characterizing treatment-induced androgen receptor (AR)-independent PC, which is a significant part of the broader category of castration-resistant tumors clinically defined aggressive-variant, previously known as anaplastic PC (2).

In addition, important recommendations regarding the assessment and interpretation methods have emerged. First, it is not recommended to routinely use immunohistochemical (IHC) stains to detect any NE differentiation in an otherwise morphologically typical primary adenocarcinoma of the prostate. In contrast, in cases of metastatic castration-resistant mixed NE-adenocarcinoma, it is recommended that the percentage and grade of the acinar component be provided. Regarding the sampling evaluation, the metastatic site, and/or the histology of the sample, most suspicious for NE differentiation should be evaluated. For confirmation of NE differentiation, markers for NE differentiation include synaptophysin, chromogranin, and CD56. Finally, the IHC expression of AR across the proposed subtypes of NEPC needs to be systematically evaluated with the aim of determining its role in classification of these tumors (2).

## Current Treatment and Biomarkers

The majority of patients with NEPC are diagnosed with locally advanced disease, usually associated with nodal or/and distant metastases. Most often, a cisplatin-based combination (e.g., cisplatin plus etoposide) has been used (3). Although the optimal regimen has not been established, most recent evidence from a phase 2 study of first-line carboplatin and docetaxel (CD) and second-line etoposide and cisplatin (EP) in 120 patients with “anaplastic” PC suggests a high response rate of short duration to platinum-containing chemotherapies, similar to small cell PC. Seventy-four of 113 (65.4%) and 24 of 71 (33.8%) were progression free after four cycles of CD and EP, respectively while median overall survival was 16 months. Intriguingly, CEA and LDH emerged as important prognostic indicators in this patient population, in contrast with tissue or serum NE markers including chromogranin A (CgA), synaprophysin, gastric releasing peptide, somatostatin, which did not predict outcome or response to therapy (4).

Plasma anterior gradient 2 (AGR2) has been proposed as a new promising biomarker for characterization, monitoring, and directing therapies for patients with metastatic NEPC. AGR2 is an epithelial marker regulated by androgens through ErbB3 binding protein 1 and Fox A transcription factors and its circulating tumor cell (CTC) mRNA and serum protein levels were found to be significantly elevated in patients with “anaplastic” cancer (5). Likewise, hASH-1 gene transcription is upregulated by androgen deprivation and is associated to the onset of an NE phenotype (6). However, a recent review of studies on the effect of NE differentiation on oncologic outcomes concludes that data are insufficient to recommend the use of NE markers in routine practice, particularly at early PC stage (7).

## Emerging Molecular Targets within the Landscape of NEPC Differentiation

A plethora of different signals involving neuropeptides, growth factors, and cytokines, are involved in the several intracellular processes including angiogenesis, cell survival, proliferation, migration, and invasion through paracrine and autocrine pathways. The resulting changes in more or less all of these processes contribute to the induction of NE differentiation in PC. The model, which has been mostly supported among several others proposed recognizes a role of previous treatment of hormone-naïve PC on the natural evolutionary transformation of classic adenocarcinoma with accumulation of new genetic alterations including loss of tumor suppressors and amplification of oncogenes (8). The finding of rearrangement of TMPRSS2–ERG in NEPC supports the origin of NEPC from prostate adenocarcinoma (8). It was thus suggested that there is an evolutionary continuum from conventional prostate adenocarcinoma to hormone naive state and finally to a CRPC/mixed state as the precursor of NEPC. NEPC has been postulated to correspond to the “cell-autonomous” phase of PC progression, which is the latest evolutionary phase following an “androgen-dependent” and a “microenvironment-driven” phase, respectively. The underlying molecular alterations at this point of progression to NEPC include loss of AR and androgen-regulated protein expression, induction of NE and neural programs, loss of tumor suppressors (TP53, RB1, PTEN) and resultant genomic instability, as well as activation of mitotic programs involving Aurora kinase A (AURKA) upregulation and MYCN amplification (5, 8).

Alternatively, adenocarcinoma and small cell carcinoma components of PC may originate from a common tumor clone with stem-like properties, or a cancer stem cell, converted into more differentiated tumor cells that accumulated molecular alterations driving epithelial or NE phenotypes. To date, very limited amounts of data are supportive of normal prostatic NE cell as a cell of origin for NE tumors (9). It is also unclear if NEPC is present at local disease or transdifferentiates later in time. Currently, as the clinical significance remains uncertain, it is not recommended to routinely use IHC stains to detect any NE differentiation in an otherwise morphologically typical primary adenocarcinoma of the prostate (2).

### Tumor suppressor loss

In both benign prostate and adenocarcinoma, the IL-8–CXCR2–p53 pathway has been shown to be a strong growth inhibitory signal that keeps NE cells quiescent (10). P53 mutation, likely a result of environmental pressure from classical and novel castration strategies inhibiting AR and intratumoral androgen synthesis, inactivates this pathway and leads to hyperproliferation and aggressive behavior of the NE cells, resulting in the development of NEPC. These findings are consistent with a clonal selection model and indicate that clones of NE cells gain a proliferative advantage in an androgen-deprived environment through P53 mutation (11).

Loss of RB1 by deletion is another common event in prostatic small cell carcinoma as Rb protein loss was found by immunohistochemistry in as many as 90% of small cell carcinoma cases (26 of 29) with RB1 allelic loss in 85% of cases (11 of 13). Interestingly, Rb protein loss rarely occurs in high-grade acinar tumors, suggesting that Rb loss is a critical event in the development of small cell carcinomas and may be a useful diagnostic and potential therapeutic target (12).

### Activation of mitosis

The resultant genetic instability leads to additional changes, many of which affect cell-cycle genes, especially those related to M-phase transition, including AURKA and Polo-like kinase 1 (PLK1). PLK1 mediates entry into mitosis as well as centrosome maturation, spindle checkpoint activity, activation of the anaphase-promoting complex, and eventual exit from the M-phase with the initiation of cytokinesis (13). LNCaP androgen-independent cells were found to have upregulation of the mitotic kinase Plk1 and other M-phase cell-cycle proteins, which rendering them highly sensitive to PLK1 inhibition through necroptosis (14).

AURKA regulates entry into mitosis, as well as assembly of the mitotic spindle apparatus, thereby affecting chromosome separation (15). MYCN amplification is frequently associated with AURKA amplification. In addition, AURKA was found to stabilize MYCN via interaction with the Skp1-Cullin-F-box (SCF)-type ubiquitin ligase FBXW7 that ubiquitinates MYCN and counteracts its degradation (16). C-MYC is also involved in NEPC and was shown to cooperate with the Proto-oncogene serine/threonine-protein kinase 1 (PIM1) in a SCID mice NEPC model, supporting the concept of targeting PIM1 (17). In line with the NEPC mitotic reprograming, in SCPC/LCNEC xenograft models, high expression of M-phase genes was found, including Ubiquitin-conjugating enzyme E2 C (UBE2C), coupled with RB and cyclin D1 loss, despite the absence of AR expression (18). A sequence of events was therefore suggested in which loss of RB and/or cyclin D1 precede AR loss and further deregulation of the mitotic apparatus.

### Epigenetic regulation changes

RE1-silencing transcription factor (REST), also known as neuron-restrictive silencer factor (NRSF) is a transcription factor that represses neuronal differentiation, in NEPC. REST-binding sites were found on 28 of 50 transcriptionally active genes in NEPC and *in vivo* in a cohort of 218 prostate tumors, in which REST downregulation was observed in 50% of NEPC tumors (19). Gene expression profiling revealed that REST not only acts to repress neuronal genes but also genes involved in cell-cycle progression, including AURKA (20).

Also intriguing was the discovery of an invert correlation between REST and the protocadherin (PCDH) genes PCDH11Y and PCDH11X (9). PCDH-PC overexpression is an early-onset adaptive mechanism following androgen deprivation therapy (ADT) and results in attenuation of the ligand-dependent activity of the AR, enabling certain prostate tumor clones to assume a more NE phenotype and promoting their survival under diverse stress conditions (21) through activation of Wnt signaling and increased nuclear beta-catenin expression (22).

In addition, downregulation of REST level relieves gene silencer REST-mediated transcriptional repression as part of a relay mechanism found in IL-6 induced autophagy through activation AMPK/mTOR pathway (23).

The epigenetic machinery involvement in NE differentiation process is a new field of ongoing research with existing data supporting a role for the inhibition of BET bromodomains in downregulation of MYC expression in PC cell lines and xenografts and more importantly “downstream” of AR (24). The histone deacetylase EZH2 is also highly expressed in NEPC and hypermethylation of key genes within the NEPC genome may be associated with the cellular plasticity seen during transdifferentiation. MYC overexpression leads to EZH2 activation by antagonizing miR-26a and PI3K–AKT-mediated EZH2 inhibition, resulting in suppression of IFNGR1 and downstream JAK–STAT1 signaling with increased cell viability and proliferation (25).

### Microenvironment changes

Acquisition of endogenous IL-6 production and its possible contribution to an autocrine cell growth stimulation may play an important role during androgen-independent progression (26). IL-6 also participates in a feed-forward loop with pigment epithelium-derived factor (PEDF) to induce NE differentiation, in which NFκB induction elicits STAT3 activation and pro-differentiating IL-6 expression causing further expansion of the NE communications (27). Activation of NFκB pathway is sufficient to maintain androgen-independent growth of prostate and PC by regulating AR action (28).

Increased paracrine release of the pro-inflammatory cytokine macrophage migration inhibitory factor (MIF) during NE differentiation in PC may facilitate cancer progression or recurrence, especially following androgen deprivation, through stimulation of AKT and ERK1/2 signaling pathways. Thus, MIF could represent an attractive target for NEPC therapy (29).

Continued focal adhesion kinase (FAK) expression (and activity) emerged as an essential factor for the androgen-independent formation of NE carcinoma in the TRAMP model (30). Targeting FAK might be an appropriate strategy in the context of arising NE phenotype in the microenvironment phase of NEPC differentiation.

Recent studies indicate the importance of the ubiquitin ligase Siah2 in control of NEPC and prostate adenocarcinoma harboring NE lesions. Siah2-dependent expression and activity of HIF-1α regulate its availability to form a transcriptional complex with FoxA2, resulting in expression of specific target genes, including Hes6, Sox9, and Jmjd1a, whose co-expression is sufficient for formation of NE tumors and NE lesions in PC. Siah is likely the best candidate, since its loss abolishes formation of TRAMP NE tumors and restoring HIF expression in such tumor cells only partially (30%) rescues formation of NE tumors. Menadione is a Siah2 inhibitor. Menadione treatment inhibited HIF levels in cultured cells, increased expression of direct Siah2 targets, and inhibited formation of melanoma xenografts. Several inhibitors directed against HIF have been recently developed. It is of importance to assess their effects in prostate tumor models (31). Hypoxia itself was shown to induce NE differentiation of LNCaP cells *in vitro*, which seems to be driven by the inhibition of Notch signaling with subsequent downregulation of hairy and enhancer of split-1 (Hes1) transcription (32).

MMP-9 produced by mast cells within the tumor microenvironment enables well-differentiated adenocarcinoma outgrowth by favoring angiogenesis and invasion to the surrounding tissue in TRAMP mice. Upon tumor progression, well-differentiated adenocarcinoma undergoes epithelial-mesenchymal transition (EMT) and foci of poorly differentiated adenocarcinoma are originated. Poorly differentiated adenocarcinoma produces MMP-9 autocrinously, thus becoming independent from mast cells. In the absence of functional mast cells (i.e., when mast cells are inhibited and/or genetically ablated), stem cell factor (SCF) in the prostatic environment is no longer sequestered and becomes available for binding to c-Kit receptor on prostate stem cells. Upon enhanced c-Kit signaling, prostate stem cells continue to proliferate, undergo NE differentiation, and progress to NE tumors, which grow fast and quickly invade the surrounding tissue. The common expression of c-Kit by mast cells and NE clones suggests a possible competition for the ligand SCF and offers the chance of curing early-stage disease while preventing NE tumors using c-Kit–targeted therapy (33, 34).

The implication of EMT in NE transdifferentiation can also occur through the effect of Snail. LNCaP PC cells transfected with Snail displayed increase in the NE markers, neuron-specific enolase (NSE) and CgA, while LNCaP C-33 cells that have been previously reported as a NE differentiation model exhibited increased expression levels of Snail protein as compared with LNCaP parental cells. Functionally, Snail-mediated NE differentiation was associated with increased paracrine cell proliferation. The novel proteasome inhibitor NPI-0052 (salinosporamide A) can inhibit Snail mRNA and protein and thereby promote sensitivity to cisplatin- and TRAIL-mediated apoptosis in DU145 PC cells. Natural products including flavonoids and parthenolide have also shown some promise toward targeting Snail signaling in PC (35, 36). Likewise, adrenomedullin, an autocrine/paracrine factor induced by androgen withdrawal, stimulates the NE phenotype in LNCaP prostate tumor cells (37).

Src kinase is another important player in the EMT process as it is activated by several neuropeptides, including CgA, NSE, serotonin, neurophysin, bombesin, and synaptophysin. Through direct physical interaction with the AR, Src is able to phosphorylate the AR and thereby induce ligand-independent AR activation (one of the key mechanisms of castration-resistant PC) (38). Neuropeptides, notably bombesin were also found to enhance early growth response 1 (Egr-1) expression leading to increased human protease-activated receptor 1 (PAR1) expression and function directly correlating with invasiveness and the degree of PC malignancy (39). Src is also a mediator for the NE-derived parathyroid hormone-related protein (PTHrP), which induces tyrosine phosphorylation and subsequent reduced AR ubiquitination thus increased accumulation of AR, enhancing growth of PC cells at low levels of androgen (40).

In addition, in co-cultures of macrophages with LNCaP and TRAMP-C2 PC cells, a feedback loop between bone morphogenetic protein-6 (BMP-6) derived from PC cells and IL-6 produced by macrophages, resulted in IL-6-induced NE differentiation in PC cells (41).

Receptor activator of nuclear factor kappa-B ligand (RANKL) either derived from the tumor or from the host plays a key role in cancer bone metastasis. A small population of RANKL-expressing cells was observed to initiate and promote cancer bone and soft tissue metastases by recruiting bystander cells to form tumors in bone. The mechanism underlying this recruitment appears to involve a feed-forward mechanism in which RANKL, RANK, and c-Met expression is increased and AR is downregulated. RANKL alters a large transcriptional program that appears to govern formation of the premetastatic niche as well as emergence of osteomimetic, EMT, and stem and NE differentiation (42). When the anti-tumor effects of the bisphosphonate zolendronic acid and somatostatin analogs (SMS) were tested on NE carcinoma models, zolendronic acid, but not SMS induced apoptosis and inhibition of proliferation and migration through impaired prenylation of Ras, thus offering the possibility of therapeutic use in the early phase for controlling NE cells (43).

The concept of “epithelial immune cell-like transition” (EIT), similar to NE-like transdifferentiation of prostate adenocarcinoma cells has been proposed to describe the acquisition of immune properties from cancer cells, which enable them to “communicate” with immune cells, leading to suppression of anti-cancer immune activity in their microenvironment and facilitation of the expansion and malignant progression of the disease (44).

Within this context, a dendritic cell vaccine sipuleucel-T was developed from peripheral blood mononuclear cells obtained by leukapheresis. In randomized trials, sipuleucel-T prolonged overall survival compared with placebo in men with minimally symptomatic, metastatic castrate-resistant PC (CRPC). However, sipuleucel-T did not affect the serum PSA, restricting this approach to patients with slowly progressive disease where a relatively rapid response to treatment is not required (45). In addition, treatment is contraindicated in patients who are on steroids or opioids for cancer-related pain, and should be used with caution in patients with liver metastases. Thus, with so far available data, assessing the impact of immunotherapy on an individual patient with NEPC can be difficult or impossible.

## Upcoming Targeted Approaches and Predictive Tools in NEPC

In the “cell-autonomous” phase of the disease, inhibitors that affect mitotic function may be efficacious, as opposed to earlier stages when AR signaling affects more “classic” AR-mediated pathways. Currently, first-line treatment for this phase is chemotherapy, but patients become rapidly resistant to this approach. As the molecular basis for NEPC becomes better understood, individualized therapy may be possible.

The AURKA inhibitor danusertib (PHA-739358) was tested in a phase II clinical trial but failed to achieve the primary endpoint of PSA response (46). However, PSA as an endpoint is unlikely to be suitable for tumors that are in the “tumor cell-autonomous” phase. In addition, therapeutic treatment in this trial was not directed specifically to patients with amplified AURKA; hence, it is not certain whether better response would have been achieved by focusing on NEPC patients with amplified AURKA (46). Also, given the enzymatic activity of AURKA depends not only on the amount of protein present but also on the activity of several cofactors (such as TPX2, BORA, and Ajuba), and has numerous substrates (including p53, BRCA1, and even AR), it is likely that the effects of AURKA (and thus the impact of its inhibition) are dependent, at least in part, on the activity of the cofactors and the role of its substrates in a given cell (47). A clinical trial evaluating the AURKA inhibitor alisertib for patients with NEPC is under way, and AURKA and MYCN co-amplification are being explored as potential predictive biomarkers and may be used to select NEPC and patients with high-risk PC for early intervention with AURKA-targeted therapy (48).

Dasatinib is a Src family/abl inhibitor with preclinical activity in PC and encouraging results in phase II studies (49). However, there was no increase in overall survival when dasatinib was given in combination with docetaxel plus prednisone compared with chemotherapy alone. In the phase III READY trial, 1522 men with metastatic CRPC were randomly assigned to either dasatinib with docetaxel plus prednisone or docetaxel plus prednisone alone. With a median follow-up of 19 months, the median survival was approximately 21 months on both treatment arms. This failure can be explained by several factors including inadequate study design, potential pharmacokinetic interactions between dasatinib and docetaxel, a stronger effect on stromal cells than on epithelial cells (despite association with the epithelial-targeted docetaxel), and the too broad specificity of inhibitory effect of dasatinib for numerous receptor and non-receptor tyrosine kinases (50).

PLK1 inhibitors have recently entered clinical trials for solid tumors. BI 2536 is a PLK1 selective inhibitor that reached phase II trial in several solid tumors, but not PC, with little efficacy (51). Volasertib (BI 6727) is a potent and relatively selective inhibitor for PLK1. A phase I study in patients with advanced disease showed a favorable pharmacokinetic profile and limited toxicities in patients with advanced solid tumors (52). Phase II studies are ongoing. On the basis of the three-phase model, PLK1 inhibitors might be effective in “cell-autonomous” tumors where PLK1 is overexpressed (8).

The role of the tumor microenvironment and data supporting MET as a potential targetable driver of NEPC was also presented as an area of active investigation (53). Cabozantinib (XL184) is an inhibitor of MET, VEGFR2, and RET. A phase II trial in patients with progressive, metastatic PC provided preliminary evidence of activity in men with bone metastases (54). Based upon these results, two phase III trials were initiated in patients who had progressed on docetaxel and either abiraterone or enzalutamide as treatment for CRPC. In the COMET-1 trial, patients were randomly assigned to either cabozantinib or prednisone; the primary endpoint was overall survival. In the COMET-2 trial, patients were randomly assigned to cabozanitinib or mitoxantrone plus prednisone and the primary endpoint was pain control. Preliminary results from the COMET-1 trial released by the corporate sponsor indicated that cabozantinib did not achieve a statistically significant increase in overall survival (55). Enrollment in the COMET-2 trial was discontinued based upon these results.

MK-2206 is an oral AKT inhibitor that was tested in a phase 1 trial using a QOD, QW, or Q3W dosing schedule in combination with carboplatin and paclitaxel, docetaxel, or erlotinib, and was well-tolerated at doses that inhibit AKT signaling. Within a diverse population of 72 patients including breast, melanoma, pancreas, prostate, colon, esophageal, small cell lung cancer, a partial response with a PFS of 6 months was shown in a patient with NEPC and minor responses were demonstrated in two patients with NE pancreatic cancers (56). Randomized phase 2 studies in specific cancer types and more homogenous cohorts are expected before being able to draw any conclusions about the clinical effects of MK-2206 with other standard cytotoxic or targeted treatment options.

Two oral endothelin receptor antagonists, atrasentan and zibotentan, have been extensively studied, to target the supporting environment for metastatic growth. Multiple phase II and subsequent phase III trials were conducted with both atrasentan and zibotentan; however, none was able to show significant benefit compared with placebo (57–61).

Given the critical roles of CTCs and EMT in PC tumorigenesis and the current immunotherapeutic strategies targeting prostate tumor antigens, such as sipuleucel-T, there may be a need to design new immunotherapies targeting cancer stem cells and cells involved in EMT.

Cumulative data on currently established and potential future targets of NEPC therapies within corresponding pathways are presented in Table 1. Most evidence for NEPC targets and corresponding targeted agents is derived from preclinical studies or *in vivo* mouse models (62–68). At the clinical level, there is no direct evidence and all data are extrapolated from studies in CRPC (50, 69–75) (Table 1). Thus, there is an urgent need for exploitation of emerging targets through design and implementation of studies in this particular subpopulation of PC patients with NE differentiated PC.

**Table 1:** **Targeted therapies in NEPC**.

Ref.	Molecular alteration	Pathway/process	Target	Agent	Result
(10, 62)	TP53 mutation	IL8-CXCR2-p53	p53–Mdm2	SAR405838	Tumor regression in LNCaP mouse model, Ph1 ongoing
(12, 63)	RB1 deletion	RB–E2F1–Mad2	Spindle disruption	Paclitaxel, STLC	Prolonged mitotic and increased cell death in PC3, DU145 cells
(14, 51, 52)	PLK1 upregulation	AURKA–PLK1–Cdc25	PLK1	BI 2536, BI 6727	Decreased proliferation and clonogenic potential of DU145, LNCaP, PC3 cells Ph1:limited toxicity and efficacy in solid tumors, Ph2 ongoing
(15, 16, 46)	AURKA, MYCN amplification	MYCN–AURKA–pH3-SYP/NSE	AURKA	Danusertib, alisertib	Ph2: 13.6% SD ≥6 mos CRPC
(17, 64, 65)	c-MYC overexpression	PIM1-c-MYC	c-Myc, PIM1	10058-F4, Quercetagetin	Reduced tumorigenic potentials of LNCaP and DU145 and reduced Pim1 protein, increased synaptophysin and Ascl1 in human PC tumors with coexpression of PIM1-c-MYC, growth inhibition of RWPE2 PC cells
(21, 22)	PCDH-PC overexpression	Wnt	PCDH-PC	PCDH-PC si-RNA	Blocked NE differentiation of LNCaP, sensitized human CRPC tumors to docetaxel
(25)	EZH2 overexpression	IFN–JAK–STAT1	EZH2	DZNep	Pharmacologic depletion of EZH2 by the histone-methylation inhibitor DZNep and synergistic antitumor effect with IFN-γ in DU145 cells
(66)	IL-6 overexpression	PEDF–NFκB–STAT3	IL-6, STAT3	Siltuximab, LLL12	Suppressed clonogenicity of stem-like cells in patients with high-grade disease and derived murine xenograft model
(29, 67)	MIF overexpression	AKT/ERK	MIF	ISO-1	Decreased tumor volume and angiogenesis in DU145 xenografts
(30, 68)	FAK overactivation	Integrin-FAK	FAK	PF-562,271, PF-00562271	Attenuation of FAK and AKT phosphorylation and abrogation of docetaxel-resistance of DU145-Rx and PC3-Rx cells, TRAMP mice
(31)	Siah2 overexpression	HIF-1α-FoxA2-Hes6/Sox9/Jmjd1a	Siah2	Menadione	Cell death by autoschizis in DU145 cells
(33, 34, 50, 69–74)	c-Kit amplification	MMP-9–SCF-c-Kit	c-Kit	Imatinib, dasatinib, sunitinib, sorafenib, masitinib, cabozantinib	Ph1imatinib: high incidence of thromboembolic events with docetaxel/estramustine combination for CRPC. Ph2 imatinib: limited PSA response, toxicities in biochemical failure patients Ph3 dasatinib: addition of dasatinib to docetaxel did not improve overall survival in mCRPC. Ph3 sunitinib: plus prednisone did not improve OS compared with prednisone alone in docetaxel-refractory mCRPC Ph2 sorafenib: 20% PR mTTP 5.9 mos, mOS 14.6 mos. Ph2 cabozantinib: improvements in bone scans, pain, analgesic use, measurable soft tissue disease, circulating tumor cells, and bone biomarkers, mOS 10.8 mos
(35, 36, 75)	Snail overexpression	NFκB–Snail–RKIP-NSE/CgA	Snail	Salinosporamide A, flavonoids, parthenolide	Inhibition of antiapoptotic gene products and chemoimmunosensitization of DU145 cells
(37)	Adrenomedullin overexpression	CRLR–RAMP2,3–NSE	Adrenomedullin	KT-5823	Inhibition of neurite outgrowth in LNCaP cells
(38, 40)	Src overexpression	PTHrP-AR	Src	Dasatinib	Ph3: addition of dasatinib to docetaxel did not improve overall survival in mCRPC
(42)	RANKL overexpression	RANKL-RANK-c-Met-AR	RANKL	Denosumab	Ph3: delayed time to first bone metastasis vs. placebo in CRPC (33.2 mos)
(43)	RAS amplification	RAS–ERK1/2	RAS	Zolendronic acid	Apoptosis and inhibition of proliferation and migration in NE allograft (NE-10) and its cell line (NE-CS), which were established from the prostate of the LPB-Tag 12T-10 transgenic mouse.
(45)	Host-cancer immunological interactions	Cellular immunity	PAP-GM-CSF	Sipuleucel-T	Ph3: sipuleucel-T prolonged overall survival (25.8 mos) vs. placebo (21.7) in mCRPC
(54, 55)	Met overexpression	HGF/Met	Met	Cabozantinib, BMS-777607	Ph2 cabozantinib: improvements in bone scans, pain, analgesic use, measurable soft tissue disease, circulating tumor cells, and bone biomarkers, mOS 10.8 mos. Suppressed cell migration and invasion of PC3 and DU145 cells
(56)	AKT overactivation	PI3K/AKT/mTOR	AKT	MK-2206	Ph1: 1 PR in NEPC

## Conclusive Remarks

The molecular characterization of NEPC is a challenging area of ongoing research with encouraging new findings on potential new targeted therapeutic approaches as well as emerging surrogate biological markers for early identification of treatment responses and failures. However, the determination of appropriate target at the right timepoint within the evolving genotype and phenotype of the disease requires constant reassessment of the underlying molecular changes. Collection and analysis of CTCs offers a great opportunity of repetitively studying these changes as a non-interventional approach compared to tissue biopsy. Ultimately, identification of appropriate targets within the signaling networks that “drive” the evolution of NEPC may not only guide development of newer biologic treatments but may also enable a more appropriate, in terms of sequencing, utilization of existing therapies to correspond to the underlying molecular biology, which dictates NEPC differentiation.

## Conflict of Interest Statement

The authors declare that the research was conducted in the absence of any commercial or financial relationships that could be construed as a potential conflict of interest.
